# Toward a Theory of Emotions in Competitive Sports

**DOI:** 10.3389/fpsyg.2021.790423

**Published:** 2021-12-16

**Authors:** Darko Jekauc, Julian Fritsch, Alexander T. Latinjak

**Affiliations:** ^1^Department for Health Education and Sport Psychology, Institute for Sport and Sport Science, Karlsruhe Institute of Technology, Karlsruhe, Germany; ^2^Department for Psychology and Education, School of Social Sciences and Humanities, University of Suffolk, Ipswich, United Kingdom

**Keywords:** emotion, competition, core affect, affect regulation, component, cycle, sport

## Abstract

In this article, we introduce a theory on the dynamic development of affective processes, affect regulation, and the relationship between emotions and sport performance. The theory focusses on how affective processes emerge and develop during competitive sport involvement. Based on Scherer’s component process model, we postulate six components of emotion that interact with each other in a circular fashion: (I) triggering processes, (II) physiological reactions, (III) action tendencies, (IV) expressive behaviors, (V) subjective experience, and (VI) higher cognitive processes. The theory stresses the dynamics of affective processes and describes the consequences for performance in competitive sports. It assumes that the peculiarities of different sports must be taken into account in order to understand the affective processes, and offers starting points on which strategies can be used to effectively regulate affective states. Consequences for research and practice are derived and discussed. To study the development of affective processes, future research should test the assumptions in ecologically valid contexts, such as real competitions or competition-like situations, using multi-component measures of emotions.

## Introduction

Sports competitions can have a deep impact on the emotional life of athletes. Because athletes often pursue personally relevant goals with uncertain outcomes, extreme states of positive and negative emotions can occur (Hanin, 2007). In fact, the emotional roller coasters in sports competitions are one of the main reasons that make sport so fascinating, both for the athletes and the spectators. Knowledge of the role of emotions during sports competitions can contribute to a better understanding of human behavior regulation in general, which in turn can serve practitioners in supporting athletes more effectively in their efforts to regulate emotions (Wagstaff, 2014).

In emotion research, a major problem lies in the often unclear use of terms, such as mood, emotions, or affect (Ekkekakis and Petruzzello, 2000). According to Russell (2003), it is important to differentiate core affect from full-fledged emotions. Core affect represents simple and rapid valuations that something is good or bad, that you like or dislike something, that you feel pleasure or displeasure. It consists of the two components valence, as a hedonic quality (pleasure vs. displeasure), and arousal, as an activation level (sleepy-activated). The core effect can be experienced as free-floating and is often associated with prototypical emotional episodes (Russell, 2003; di Fronso et al., 2020). It represents a continuous assessment of one’s current state, which is outside the person’s awareness most of the time. However, a person can become conscious of it when attention is drawn to it. In this sense, core affect is constantly running in the background, changing in response to internal processes or changes in the environment (Russell, 2015). Russell (2009) defines core affect as “a neurophysiological state, accessible to consciousness as a simple non-reflective feeling” (p. 1264). Thus, core affect can be understood as a state that is at the heart of emotions (di Fronso et al., 2020) and the starting point for emotion development.

From core affect, as a rather quick valuation of a situation, a wide range of full-fledged emotions saturated with cognitions can develop (Weiner, 1986; Baumeister et al., 2007). A full-fledged (sometimes also called full-blown) emotion is a progression of core affect that typically involves physiological arousal and emotion expression, is reflected in a consciously experienced feeling, and is associated with consciously experienced higher cognitive processing. According to Baumeister et al. (2007), a full-fledged emotion arises and dissipates only slowly compared to the core affect. Its main functions are to direct attention and stimulate reflection in order to learn the necessary lessons from the situation. In this way, people learn to adapt to their environment. As a consequence of a deeper elaboration of the situation, more elaborated and differentiated emotions (e.g., anger, anxiety, joy) can be consciously perceived by the individual. Consistent with Clore and Ortony (2008), we use the term “affective processes” when referring to processes including core affect and/or differentiated emotions.

Sport psychology has a long history of component theories^1^ of emotion that describe the relationship between emotions and performance. For example, Yerkes and Dodson (1908) emphasized the relevance of the physiological component, postulating an inverted u-shaped relationship between arousal and performance. Within the framework of the multidimensional anxiety theory, the model was extended in order to include the cognitive component, with worry being a cognitive form of apprehension (Martens et al., 1990). According to the multidimensional anxiety theory, cognitive anxiety has a negative linear relationship with sport performance, while somatic anxiety has an inverted-U relationship. By explicitly considering the interaction between the two anxiety components, the catastrophe model of anxiety suggests that the influence of somatic anxiety depends on the level of cognitive anxiety (Hardy, 1990). Accordingly, when cognitive anxiety is low, somatic anxiety has a small effect on sport performance in the form of an inverted-U. In contrast, when cognitive anxiety is high, somatic anxiety enhances sport performance up to a critical point. However, when this threshold is surpassed, a further increase in somatic anxiety leads to a catastrophic drop in performance. While one meta-analysis indicates that neither cognitive nor somatic anxiety are related to sports performance (Craft et al., 2003), another meta-analysis shows a weak negative relationship between cognitive anxiety and sport performance (Woodman and Hardy, 2003). More recently, Cheng et al. (2009) proposed a third separate component involving a regulatory aspect of anxiety. Finally, Carson and Collins (2016) introduced a fourth component, which focused on motor aspects and the self-confidence associated with them. In addition to the inconclusive evidence and theoretical disagreement, the exclusive focus of these theoretical approaches on anxiety has often been criticized (e.g., Sève et al., 2007; Martinent and Ferrand, 2009; Uphill et al., 2014).

The Individual Zone of Optimal Functioning (IZOF) model goes beyond anxiety and focuses on functional emotions with a positive impact on athletic performance and dysfunctional emotions with a negative impact on athletic performance (Hanin, 2007). Based on an idiosyncratic approach, the IZOF model suggests that athletes should determine their own zone, considering a maximum amount of functional and a minimum amount of dysfunctional emotions. While the IZOF model seems to have great applied value, it cannot explain the mechanisms of how the individual zones influence athletic performance (Woodman and Hardy, 2001). Finally, another theory that has gained increasing attention in sport psychology is cognitive-motivational-relational theory, assuming that each specific emotion has a core relational theme with a related action tendency (Lazarus, 1991). With regards to sport performance, it is assumed that the action tendency for each specific emotion can increase sport performance when it suits the demands of the situation and the needs of the athlete (Lazarus, 2000). For instance, the action tendency “lashing out” of the emotion anger appears to be beneficial in sports that require aggression and gross motor movements, and detrimental in sports that require precision and fine motor movements. Cognitive-motivational-relational theory has been useful to explain why emotions occur (Uphill and Jones, 2007; Lewis et al., 2017) and how they in turn affect sport performance (e.g., Woodman et al., 2009; Rathschlag and Memmert, 2015). From our point of view, however, the theory has not been able to contribute decisively to explaining the dynamics of affective processes in the sports context. As emotions show enormous fluctuations (Vallacher and Nowak, 1997), the aspect of dynamics plays a crucial role in understanding affective processes, especially in the context of sports.

Overall, emotion theories used in sport psychology have two shortcomings that we aim to overcome in this paper: they refer to anxiety, and the conceptual delimitations between emotion and core affect are not clearly defined. In sight of these shortcomings, we assume that research on emotions in sport would particularly be beneficial when considering explicitly (a) the distinction between full-fledged emotions and core affect (Ekkekakis and Petruzzello, 2000), (b) the multi-componential nature of emotions (Scherer, 2005), and (c) the dynamics of affective processes in sports. In this article, we present a new theory of emotions by addressing three issues: First, we present the component model of emotions in sports, elaborating on how affective processes develop into emotions. Second, we discuss the dynamics of affective processes and their potential effects on sports performance. Third, based on our model, we outline suggestions of how affective processes can be regulated.

## The Component Model of Emotions in Sports

The theoretical framework presented in this article is based on the five emotion components suggested by Scherer (2005): the cognitive component, neurophysiological component, motor expression component, motivational component, and subjective feeling component. Extending Scherer’s position, we argue that cognitive processes occur in two stages within the emotion generation process and, thus, can be separated into two distinct components. The first component refers to simple, rapid, and automated cognitive appraisal processes relevant at the very beginning of the emotion process. In line with this assumption, Moors and De Houwer (2001) were able to show in an experimental study that stimulus valence and motivational relevance as two relevant appraisal proccesses can be determined quickly and automatically. The second component refers to higher cognitive processes, such as analyzing, planning, or making decisions, and is relevant at a later stage in the emotion generation process. This distinction of the cognitive processes into two components also coincides with the position of dual process theories (Clore and Ortony, 2008), and shows similarities with the concepts of primary and secondary appraisal (Lazarus, 2001). Regarding neurophysiological aspects, LeDoux (1998) also distinguished two pathways of emotion processing. Quick and simple appraisals could be made on the low road, and more complex and differentiated representations of the situation on the high road.

**FIGURE 1:**
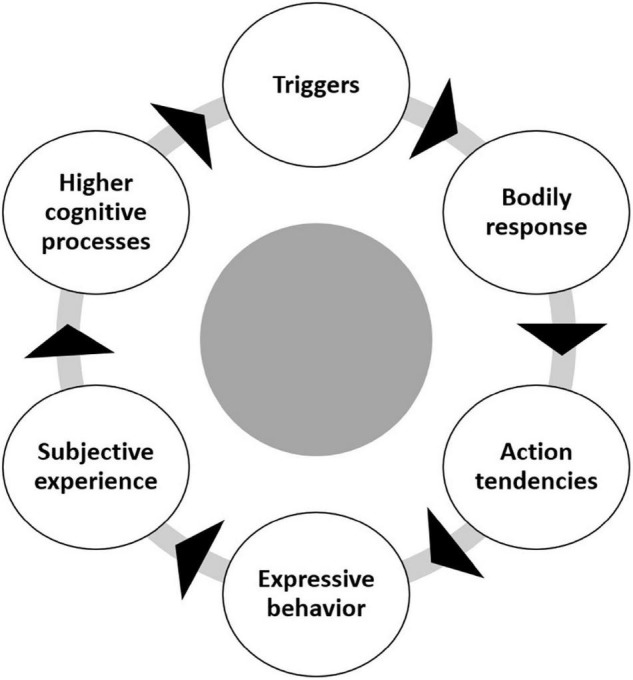
Six components in the cycle of emotions.

Taking into account the differentiation of simple and rapid cognitive appraisal processes and higher cognitive processes, the main assumption of the model is that the affective processes can be described as a cyclical mechanism (Cunningham et al., 2015) comprising six components (Figure 1). Affective processes begin with a stimulus that interacts with internal states, which can be represented as *triggering processes*. The triggering processes can lead to *physiological reactions*, *expressive behaviors*, and *action tendencies*. These, in turn, can lead to a *subjective experience* and a stimulation of *higher cognitive processing* potentially resulting into a state of full-fledged emotion. Although it is assumed that changes in one component lead to changes in subsequent components, operations in one component do not have to be fully completed before changes are initiated in subsequent components. Rather, processes in the respective components run at least partially parallel to each other (Scherer, 2001). Moreover, the changes triggered in the subsequent components may also feedback to the previous components in the next loop of the cycle of emotions. We understand these recursive and iterative processes as the cycle of emotions. In this view, affective processes can be seen as an ongoing interaction of components that produces emergent properties not fully derivable from the component states (Coan, 2010).

### Triggering Processes

Affective processes are initiated by internal or external triggers (Izard, 1993). While internal triggers refer to internal processes (e.g., thoughts, feelings, physiological processes) without a direct input from the outside, external triggers are related to environmental stimuli. Relevant environmental stimuli can be applied to many different kinds of situations directly associated with the sports competition (e.g., winning or losing, behavior of opponent), but can also go beyond the sports competition (e.g., family situation, playing for a new contract; Hanton et al., 2005). At the same time, certain environmental conditions may be relevant only to a particular sport, such as the waiting time before executing a jump in pole vault or the scoring system in table tennis (Sève et al., 2007). Although it is possible to show that such situational factors can influence affective states in a probabilistic way (e.g., Moesch et al., 2015; Fritsch et al., 2020a), it is not the stimulus itself but the way the individual appraises the stimulus that elicits affective states (Lazarus, 1991; Scherer, 2001). The way an individual appraises a situation at a given moment depends on various dispositional (e.g., trait optimism, emotional intelligence, personal values) as well as more transient personal factors, such as state self-confidence, expectations, the current affective state, etc. For instance, studies within the sports context have shown that a high emotional intelligence is associated with a lower cortisol response (Laborde et al., 2014) and a better athletic performance (Kopp and Jekauc, 2018), or that a high self-efficacy is related to more functional and less dysfunctional biopsychosocial states (Di Corrado et al., 2015). As a result of the interaction between person and situation, we assume that the most relevant proximal appraisal categories within the sports context are *significance* and *goal-related expectancies*.

A situation has to be appraised as significant for one’s own needs, values, or current goals to elicit affective processes (Frijda, 1988). In sports, the significance of a situation is typically higher the more consequential the outcome of the situation is (McGregor and Abrahamson, 2000). For instance, table tennis players are more likely to show expressive behavior in elimination matches than in group stage matches (Fritsch et al., 2020a). With relevance for negative emotions, the perceived significance of an upcoming event can predict the intensity of cognitive and somatic anxiety (Lane et al., 1995). Furthermore, it is important to consider the idiosyncratic nature of the appraisal process (Lazarus, 1991). A player might appraise a match as significant, although the outcome of the match has no impact on the ranking (e.g., when playing against the old team in an unimportant competition).

It is very natural for athletes to have goals and goal-related expectancies about their likely performance or the outcome of a competition (Uphill and Jones, 2007). Expectancies are beliefs about a future state and can be regarded as standards or reference values (Roese and Sherman, 2007), which are used to compare the current situation with the expected situation, concerning the progress toward one’s goals (Carver and Scheier, 2001). In accordance with Carver and Scheier (1990), we argue that athletes’ *affective states* (core affect) change as a result of the comparison between the expected rate of progress and current rate of progress toward the goal. Due to the dynamic nature of a sports competition, athletes in many sports receive continuous feedback on the progress toward their goal achievement (e.g., in form of a score). The progress toward one’s goal is evaluated on an ongoing basis. If progress is moving in the right direction, positive affective states occur. In contrast, when progress toward one’s goal is threatened, negative affective states occur (Carver and Scheier, 1990). Within the context of competitive sports, it was found that exceeding goal-related expectations can generate positive affective states and not reaching these can generate negative affective states (McGraw et al., 2005). In this model, we argue that in sports the appraisal of significance (e.g., important match situation) and goal-related expectancies (e.g., unexpectedly poor performance) are typically the starting point for further affective processing and can induce physiological reactions.

### Physiological Reactions

The initiation of affective processes lead to physiological reactions elicited in the autonomic (Kreibig, 2010) and central (Davidson, 2003) nervous system. These physiological reactions (e.g., change in heartbeat, skin conductance, dilation of the pupil, etc.) prepare the organism to cope with the demands of the situation (Mendes, 2016). In line with LeDoux’s (1998) distinction between the low and high pathways of affective processes in the brain, we argue that the low road is particularly related to appraisals of relevance and goal-related expectancies. Thus, in case a stimulus is appraised as significant, on the low road, information about the stimulus will directly reach the amygdala through the sensory thalamus. The amygdala then initiates an endocrine reaction associated with an activation of the sympathetic nervous system. The associated physiological reactions prepare the body for a rapid response. This rapid response has been an evolutionary advantage; however, the information about the stimulus is a rather rough representation of the stimulus (LeDoux, 1998). There are various parameters that can be used to assess the physiological reactions such as heart rate, muscle tone, skin conductance, dilation of the pupil, blood pressure, cortisol, etc.

On the high road, information about the stimulus is forwarded from the sensory thalamus to the cortex, where a more thorough analysis of the stimulus is performed using higher cognitive processes. These higher cognitive processes then influence subcortical regions such as the amygdala and the associated physiological reactions (Ochsner et al., 2012). Cunningham and Zelazo (2007) suggest that such processes are rather iterative, a kind of “evaluative circle” in which stimuli are interpreted and reinterpreted in the light of increasing situational representation. In the early stages, when the evaluations are based on few iterations, the process is rather unconscious and automatic. With an increasing number of iterations, the process is more and more consciously accessible (Cunningham et al., 2015). The high road is related to the processes described in the section “Higher Cognitive Processes.”

### Action Tendencies

In the course of the emergence of the physiological reactions, an action tendency develops, which is a state of readiness to perform a certain type of action (Frijda, 1986). The organism is getting prepared to act immediately according to its affective state. This component represents a motivational aspect and refers to an urge to carry out certain expressive behaviors associated with the affective state. It is important to emphasize that action tendencies are always embedded in a situation and can only be expressed in the context of that situation (Frijda, 1986). Action tendencies reflect some action modes, such as attacking or defending, which can manifest at different levels (e.g., interpersonal behavior, tactics of the match, etc.). Action tendencies should be considered as a separate component from expressive behaviors, since individuals always express their emotions more or less subtly (Frijda, 1986). However, action tendencies are not always translated into behavior and can only be inferred from physiological measurements in the brain (e.g., readiness potentials, Cunnington et al., 2003; Nguyen et al., 2014). Therefore, a direct measurement of action tendencies in the context of physical activity and sport is hardly possible.

### Expressive Behaviors

Physiological reactions and action tendencies are closely related to expressive behaviors (Tomkins, 1962). We argue that internal physiological states have a tendency to be externalized in form of facial expressions, gestures, postures, movements or verbalizations. The higher the level of physiological arousal, the more likely expressive behaviors will be shown (cf. Banse and Scherer, 1996). According to Darwin (1872), expressive behaviors have additional adaptive functions, such as sending social cues to teammates and opponents, which can be expressed automatically as well as intentionally manipulated for strategic reasons. Although there is a great debate about the universality of body channels to display emotions (Barrett et al., 2019), observers can generally draw accurate inferences about the valence of an individual’s affective state (Furley and Schweizer, 2014, 2016). However, studies have emphasized that the integration of different body channels provides better recognition rates than the use of a single channel (Aviezer et al., 2012a,b). One method to assess this component would be direct observation, which has already been successfully implemented in some sports, such as handball (cf., Moesch et al., 2015, 2018).

### Subjective Experience

If the intensity of the previous processes is strong enough, the individual becomes aware of the affective processes and can report them verbally (Scherer, 2009). The subjective experience is often considered as the most essential component of emotions, which distinguishes emotions from other psychological states and, in turn, directs further cognitive processes (Scherer, 2009). This stage of affective processes represents the transition of an automated and unconscious affective state to an increasingly conscious state of a developing emotion. Interestingly, a considerable amount of research has shown that negative aspects of a situation have a stronger effect on the subjective experience than positive ones (Baumeister et al., 2001). According to Frijda (1988), this hedonic asymmetry, which represents the tendency to become more difficultly accustomed to aversive circumstances compared to pleasant ones, is hardwired in the human brain and a product of an evolutionary development. For the context of competitive sports, hedonic asymmetry means that negatively valuated events have a higher probability to influence the subjective experience than positively valuated events. Because athletes often report that they struggle with negative emotions (Martinent et al., 2015), this points to the importance of appropriate affect regulation skills. Numerous questionnaires such as Sport Emotion Questionnaire (Jones et al., 2005) or Competitive State Anxiety Inventory-2 (Cox et al., 2003) or qualitative interviews with athletes are available to measure this component.

### Higher Cognitive Processes

The last component in the cycle of emotions refers to higher cognitive processes, such as causal reasoning, systematic decision making, higher cognitive control processes or planning (Ochsner and Gross, 2005). These processes should not be confused with the simpler and more automated cognitive processes that occur in the preceding emotion components. From this point on, a specific emotion can be perceived and one can refer to an *emotional state* instead of an affective state. After athletes become aware of their affective state, they begin to search for an explanation for their own feelings (Jones, 1995). Athletes might ask themselves: Why do I feel like this? Rather, complex analyses of the situation and one’s own feelings are initiated, which comprise the appraisal of the own resources (e.g., control), the agent responsible (self vs. other), implications of the rate of progress toward achieving own goals (e.g., score in the match) as well as social standards (e.g., violated vs. not violated). According to Weiner (1985) as well as Ortony et al. (1990), such appraisals can lead to full-fledged emotions (e.g., anger, anxiety, pride). For instance, when becoming aware that the opponent’s behavior deviates from established rules (e.g., cheating), or that unwritten norms (e.g., celebrating when the opponent makes a mistake) can lead to differentiated emotional states (Lewis et al., 2017; Lee et al., 2018). In general, it has been shown in sports that such more complex appraisals can contribute to the development of concrete and distinct emotions (Uphill and Jones, 2007; Uphill et al., 2014).

These appraisals can lead to the emergence of new thoughts, which in turn can induce a new cycle of emotions. In this sense, thoughts can act as an inner trigger that starts the circular process from the beginning resulting in a continuous change of emotions. For example, a thought about the reasons for the course of the match may induce a cascade of new thoughts that are associatively linked to each other. In the long chain of these associatively linked thoughts, however, the sixth- or seventh-order thought might induce a new emotional state that might not be directly related to the original emotional state. In this way, a new emotional state can also be induced without a direct input from outside.

## Cycle of Emotions and Performance in Sports

**FIGURE 2:**
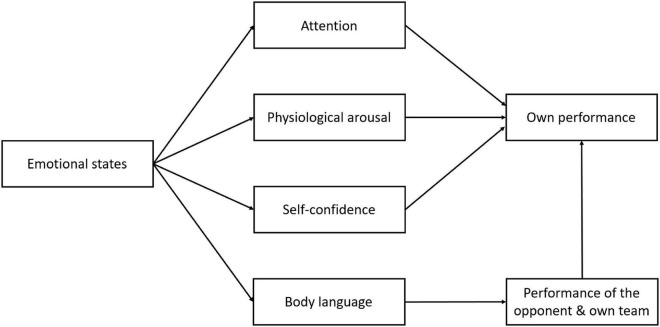
Mechanisms of influence of affective states on performance in sport.

All six components represent different aspects of affective processes and provide information as to how they can be measured. The interconnectedness of the components and the cyclical nature of affective processes create a dynamic that can, in various ways, influence psychological processes relevant to sport performance. The relationship between emotions and performance in competitive sport appears to be complex (Lazarus, 2000) and it is likely to be reciprocal (Buenemann and Schweizer, 2021). On the one hand, as shown above, the current performance and outcome-related events within the competition can influence the triggering processes of an emotion. On the other hand, emotional states might influence performance of the athlete. However, we believe that there is no universal relationship between emotional states and performance that applies to all sports. Rather, it depends on the physical and mental demands of the specific sport. For example, some sports require high levels of force (e.g., rugby, Robazza and Bortoli, 2007) or even aggression (e.g., boxing, Martinez, 2017), whereas other sports (e.g., darts) require more precise movements and sustained attention. The heterogeneity of results in relation to the association between emotional states and performance in different sports, as shown in numerous studies (cf., Woodman and Hardy, 2003),suggests that emotional states do not affect performance *per se*, but may influence other physiological and psychological processes that in turn affect athlete performance. Based on the different emotion components of the model described above, we derive that there are least four possible mechanisms as shown in Figure 2.

### Effects on Sport Performance *via* Cognitive Component and Attention

First, we hypothesize that emotional states can have an indirect effect on performance *via* attention. Emotional processes are supposed to attract attention (Baumeister et al., 2007), which is considered a core competency for athletes to perform at their best (Moran, 2016). Pressure situations associated with negative emotions (e.g., anxiety) are thought to impair athletes’ performance by drawing attention to thoughts irrelevant to the task, such as worries about the situation and its consequences (Wine, 1971; Sarason, 1984). These consequences may then elicit efforts to focus attention on relevant aspects of the competition and to suppress accelerating thoughts and action tendencies. Such self-regulatory activities consume cognitive resources that are limited and can be depleted (Eysenck and Calvo, 1992; Baumeister et al., 1994). Because maintaining attention and suppressing thoughts are assumed to use the same cognitive resources (Baumeister et al., 2000), emotionally demanding sport involvement can lead to a state of cognitive exhaustion and a breakdown of attention. This idea is consistent with the cognitive load hypothesis (Mitchell and Phillips, 2007), which states that affective states lead to rumination and therefore reduction in cognitive capacity, which in turn influences athlete’s performance. In this vain, we assume that such drops in performance are more likely to occur after emotionally exhausting periods of competition. In the same vein, pressure situations associated with negative emotions can increase self-consciousness and thus foster the phenomenon of choking under pressure. According to Baumeister (1984), in pressure situations, athletes tend to focus their attention on executing the well-learned components of a complex, proceduralized movements (e.g., putting in golf), thereby disrupting automatic movement execution. Both the self-focus and distraction hypotheses have received ample empirical support (Mesagno et al., 2015) and it appears that both mechanisms are relevant to the effect of attention on performance as a function of sport demands. In relation to our consideration of the components of emotion, the attention mechanism refers mainly to the component of higher cognitive processes and indicates how these may affect athletic performance. In our model, the affective processes prompt an analysis of the situation that demands attention and other cognitive resources. Thus, through this analysis, distraction from the actual task occurs and attention is used to consciously control the well-learned movements, eventually depleting cognitive resources and disrupting movement performance.

### Effects on Sport Performance *via* Physiological Arousal

Second, as described above, emotional processes are always associated with physiological reactions (e.g., increase in heart rate, increase in muscle tone). which in turn may have an impact on performance in sports. Early on, it was speculated that physiological arousal has an effect on motor performance and that the relationship can be represented in terms of an inverted U (Yerkes and Dodson, 1908). One possible explanation is that the increase in physiological arousal leads to an increase in muscular tension, which in turn, above a certain level, has negative effects on motor performance (Courts, 1942). A large body of empirical evidence suggests that a relationship exists between physiological arousal and motor performance (Parfitt and Pates, 1999), whereas the form of this relationship is not fully understood (Arent and Landers, 2003; Landers, 2007). Another pathway of action could be that physiological arousal has an impact on performance *via* cognitive processes (Lautenbach and Laborde, 2016). However, due to the large heterogeneity of the tasks focused, their results cannot be generalized to all sports. Our conjecture is that the relationship between physiological arousal and performance in competitive sports depends on the demands of the sport in question. While a low arousal level is optimal for sports with high relevance for precision (e.g., sport shooters), a high arousal level is conducive to performance in sports with a high power component (e.g., rugby).

### Effects on Sport Performance *via* Expressive Behavior

Third, expressive behaviors, as central component of our model, can be viewed as social signals to teammates or opponents. A number of studies in various sports have shown how non-verbal behaviors can influence observers’ self-efficacy, expectations, and emotions (Greenlees et al., 2005a,b, 2008; Furley and Schweizer, 2014; Furley et al., 2015; Seiler et al., 2018). Importantly, the direction of such effects depends on the relationship between the individuals expressing the emotion and the observer. Whereas negative expressive behaviors (e.g., frustration) tend to increase self-efficacy and elicit positive emotions in out-group members, they tend to decrease self-efficacy and elicit negative emotions in in-group members, and vice versa for positive expressive behaviors (Furley et al., 2015; Furley and Schweizer, 2020). Given these findings, it is not surprising that athletes report deliberately using expressive behaviors to gain an advantage in the “psychological battle” within a competition (Fritsch et al., 2021). Therefore, we assume that an athlete’s emotional expressive behavior indirectly affects his or her own performance by influencing the performance of opponents and teammates.

### Effects on Sport Performance *via* Self-Confidence

Forth, affective processes are postulated to influence athlete performance *via* self-confidence. In the sports context, self-confidence has been defined as the degree to which athletes are certain about their ability to be successful (Vealey, 1986). In this conception of self-confidence, which focuses on the certainty of success and the evaluation of one’s own abilities, it seems plausible to assume that affective processes play also an important role. Some emotion theories, such as Scherer’s component theory of emotion or Ortony’s structural model of emotion, assume that appraisal processes related to certainty of outcome and responsibility for outcome play a central role in the emergence of emotions (Ortony et al., 1990; Scherer, 2009). This reasoning implies that self-confidence and emotional states are very closely connected due to the common appraisals in the formation process of the two constructs. In support of this argument, a number of studies have shown that self-confidence and emotional states (e.g., anxiety) have a moderate to high negative correlation in sports (e.g., Martens et al., 1990; Sklett et al., 2018; Dallas et al., 2019). The relationship between self-confidence and performance in sport is also well documented, with results from a meta-analysis suggesting moderate effects (*r* = 0.24) of state self-confidence on performance in competition (Woodman and Hardy, 2003). As these results illustrate, self-confidence as an emotion-like construct has a stabilizing effect on performance in sport. Thus, self-confidence can be seen as a counterpart of negative emotions such as anxiety, which, as we will see below, tend to have a destabilizing effect.

### Development of the Relationship Between Affective Processes and Athletic Performance Over Time

**FIGURE 3:**
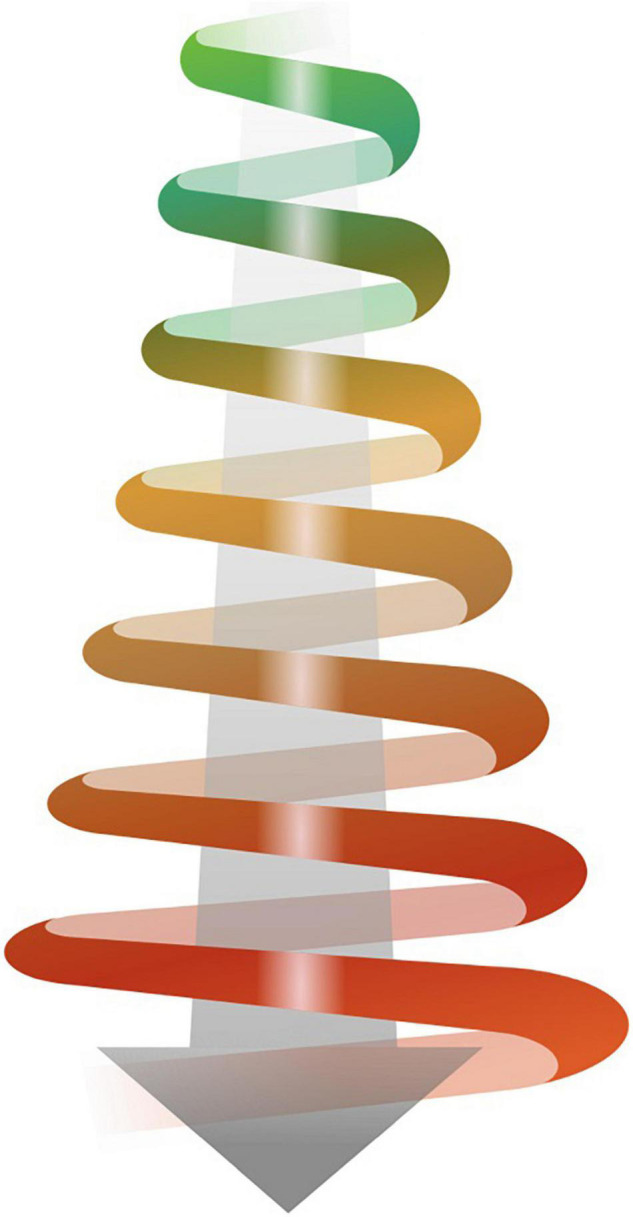
Spiral of emotions.

As could be illustrated above, research shows that the effects of affective states on athlete performance manifest at multiple levels. Looking at the relationship over time, the relationship between affective states and performance is dynamic and non-linear due to the cyclical mechanisms explained above. It is assumed that an athlete can perform more or less at a constant level as long as he or she is in a constant affective state. Conversely, abrupt changes in affective states could lead to abrupt changes in performance. In this way, affective processes within individual matches can cause abrupt turns in the course of the match, causing unexpected and improbable developments. These developments can perhaps best be represented by the metaphor of a spiral, in which the development of a match is accompanied by the development of affective states (see Figure 3). For example, a simple mistake in an *important situation* of a tennis match can be the starting point for affective processes (*triggering processes)*. The mistake is *unexpected*, because the player rarely hits the ball into the net from a position close to the net. The appraisal of an unexpected error in an important situation of the match can cause *physiological reactions* that lead to an increase in *arousal*. The player expresses his frustration by shouting loudly and gesticulating desperately *(expressive behaviors)*. This, in turn, can boost the self-confidence of the opponent, who now senses a chance to turn the match around. The player might now feel an inner urge to throw the tennis racket on the ground (*action tendency*) and becomes *aware* of the anger (*subjective experience*). Instead of giving in to the *anger tendency*, he begins to *analyze the situation* and *look for the reason* for the mistake (*higher cognitive processe*s). He might reason that he normally never makes such simple mistakes and that the surface of the court is uneven, making it impossible to play tennis at his normal level. He might ask himself how to deal with this difficult situation. In the midst of these *thoughts*, the player has to begin with the next point. Lacking full attention, the player misses his first serve and loses the point. The cycle of emotions enters the next loop and the player sinks deeper and deeper into the spiral of negative emotions. Consequently, the player loses several points in a row and gets into a seemingly miserable situation that could not have been foreseen in the previous stages of the match.

This example illustrates what such a cycle of emotions might look like in tennis. While this example suggests that the loss of attention is a central link between emotions and performance, it is important to emphasize that there are also other relevant mechanisms as described above. The extent to which the cycle of emotion unfolds its full power and how strongly it influences the athlete’s performance depends largely on their affect regulation capacities.

## Affect Regulation Within the Cycle of Emotions

As mentioned above, affect regulation capabilities play a crucial role in understanding the relationship between affective states and performance in sports. When affect regulation strategies are properly used, we can put ourselves in affective states that enable us to perform at our best. It is important to note that more than 400 specific affect regulation strategies have been identified (Augustine and Hemenover, 2009; Koole, 2009) so far, and some of them have been thoroughly discussed in sport psychology (Uphill et al., 2009). In the following, we want to discuss the role of different affect regulation strategies within the cycle of emotions.

Consistent with our distinction between core affect and emotion, in our theory we use the term affect regulation instead of the term emotion regulation, which is commonly used in sport psychology. Affect regulation includes the regulation of all affective processes, including those processes that cannot yet be described as being emotional (e.g., regulation of appraisals). In accordance with Campos et al. (2004), we assume that the development of affective processes and affect regulation cannot be clearly separated from each other. Rather, affect regulation begins as early as the affective processes themselves (Gross and Thompson, 2006) and manifests differently in the various components. Hence, affect regulation is a dynamic process which allows a versatile response to the challenges of the environment by dampening or intensifying the different components. This means that affect regulation strategies can be applied to any component of the cycle of emotions. Although many affect regulation programs target multiple components to optimize effectiveness (e.g., Kittler et al., 2018), the focus is usually set on one particular component. For this reason, assigning the different strategies to the different components of the cycle of emotions provides a sound basis for classification.

This new taxonomy of affect regulation strategies diverges from Gross’s (2014) currently prevailing model of emotion regulation, which primarily assigns the individual strategies to the timing of affective process. Moreover, we assume that certain phases of affect regulation within Gross’s model, such as situation selection, have little application within the context of sports. At the same time, other strategies, such as reflecting on one’s own expectations within the component “triggering processes,” play an important role in regulating affect in athletes. Therefore, we introduce a new taxonomy of affect regulation strategies classified in accordance with the components of the cycle of emotions.

First, affect regulation may refer to the regulation of triggering processes. In the context of our theory, this means that an athlete might regulate affective states by influencing the appraisal of the significance of the situation and by reflecting on one’s own expectations, goals, and standards. The importance of these two factors was also demonstrated in a qualitative study by Hill and Shaw (2013) in which all athletes who experienced the phenomenon of choking under pressure reported that the significance of the situation and expectations were important determinants of pressure and negative affect. In this sense, downplaying the importance of the match (Berendt and Uhrich, 2018) and reducing one’s own importance as well as public expectations (Hodge and Smith, 2014) are commonly used strategies to reduce negative affective states in competitive sports. Regarding sport performance, some studies have also shown that manipulation of appraisals increased athlete performance (cf., Rumbold et al., 2012).

Second, for the physiological reactions component, affect regulation means influencing the body’s physiological arousal. Thus, relaxation techniques, like progressive muscle relaxation, are considered one of the most common strategies for regulating one’s arousal level and thus one’s own affective states (Lane et al., 2012). In some studies, relaxation has been shown to be an effective strategy for reducing competitive sport anxiety (e.g., Fletcher and Hanton, 2001) and regulating athletes’ affective states (e.g., Hashim et al., 2011). Again, regarding sport performance, several studies show that relaxation as a strategy to influence the physiological states can lead to improved performance (Weinberg et al., 1981; Madden and McGown, 1988; Kachanathu et al., 2013).

Third, the regulation of action tendencies is an important aspect of affect regulation in sport, as the intensity of one’s actions has a significant impact on performance. Strategies for a controllable conversion of action tendencies into appropriate and purposeful actions, therefore, represent an important factor in the development of the abilities to regulate emotions. In dealing with anxiety, acceptance-based approaches to regulate action tendencies have been shown to be more effective than mere emotion suppression (Roemer et al., 2008). Within the context of sport, the Mindfulness-Acceptance-Commitment approach (Gardner and Moore, 2007) has laid the foundations for countering action tendencies with acceptance and commitment toward actions consistent with performance and personal values. A number of studies showed that such forms of training also lead to improvements in performance (Gardner and Moore, 2020).

Fourth, it is not only that affective states have a tendency to be externalized, but also the way in which affective states are expressed which influences subsequent affective processes. For the component of expressive behavior, affect regulation means influencing one’s own expressive behavior, as long as it is under one’s own control. A number of studies have found that consciously holding a body pose is associated with changes in self-esteem (e.g., Ramezanzade and Arabnarmi, 2011; Körner et al., 2019) and expectancy of success (Furley et al., 2012). In fact, it seems that by regulating one’s expressive behavior, one implicitly regulates various psychological parameters, such as one’s self-confidence and attention (Fritsch et al., 2021).

Fifth, regarding the subjective experience, becoming aware of one’s own affective states seems to be an important step toward successful affect regulation (Ludwig and Kabat-Zinn, 2008). By becoming aware of one’s affective states, one is able to face them with a certain serenity. This serenity, in turn, may help to reduce the intensity of affective states. Based on this idea, several emotion-focused approaches for affect regulation have since been developed (Greenberg, 2011; Leahy et al., 2011). Within the sports context, similar programs have been developed to improve affect regulation (Kaufman et al., 2009; Jekauc et al., 2016). Along these same lines, it has also been shown that mindfulness training, which emphasizes awareness of one’s affective states, leads to improvements in affect regulation skills in athletes (Kittler et al., 2018). It has also been shown that athletes who actively perceive and accept their emotions and affective states are more able to cope with pressure and are less prone to choking under pressure (Hill et al., 2010; Hill and Shaw, 2013).

Finally, emotions can also be regulated with the help of higher cognitive processes, such as imagery of desired states, setting and pursuing goals, or controlling thoughts. The regulation of higher cognitive processes has a long tradition in cognitive behavioral therapy (Beck, 1976) as well as general psychology (Ochsner and Gross, 2005). Many of these practices have been used successfully in sport psychology, for example, imagery (Lane et al., 2012), goal-setting (Adie et al., 2008), self-talk (Uphill et al., 2009; Fritsch and Jekauc, 2020), or pre-performance routines (Mccann et al., 2001; Malouff et al., 2008; Mesagno and Mullane-Grant, 2010; Hazell et al., 2014; Lautenbach et al., 2015). In general, these cognitive techniques have been shown to be effective for improving performances (Jones, 2012).

This new taxonomy of affect regulation has some new implications for the sport context. In accordance with Gross (2014), we assume that affective processes in its emergence can be controlled much more easily than when an emotion is already fully developed. To prevent the dynamics of affective processes from arising in the first place, the early components, especially the triggering component, could be focused on more strongly. Thus, in the practice of competitive sports, it is also frequently observed that athletes and coaches try to lower the goal-related expectations and significance of the competition in interviews and speeches prior to a match in order to reduce the likelihood of an affectively charged situation occurring. This procedure can be understood as an intuitive strategy for affect regulation. Therefore, the cycle of emotions offers a somewhat different perspective on how emotions arise and how they can be regulated. Thus, it is clear that affective processes can be influenced within several components simultaneously.

From a sport psychology perspective, it is important to note that the processes associated with the first two components (i.e., triggering processes and physiological reactions) occur on the rather unconscious level and are not under volitional control. For example, athletes may feel positively or negatively aroused without knowing the reasons for their state. This automatic arousal could also explain why athletes often have difficulties regulating their affective states even though they are actively trying to do so (Martinent et al., 2015). For affect regulation, this means that these early affective processes cannot be directly influenced, and affect regulation strategies related to these components should be trained on a long-term basis (e.g., relaxation strategies, mindfulness). For example, the regulation of physiological states can be learned by training relaxation. Normally, an athlete can only apply relaxation strategies during competitions after he or she has practiced the method for several weeks or even months (Pineschi and Di Pietro, 2013). Regarding mindfulness, athletes can learn to understand which appraisals imply affective states. Understanding and accepting one’s own affective states are a long-term process (Blackledge and Hayes, 2001).

Affect regulation in the later components of the cycle of emotions (i.e., subjective experience, higher cognitive processes) tends to occur on the high level of affect processing and is more subject to conscious processes and volitional control. Volitional control over one’s own affective states can be very effective and gives a good feeling of being in control of the situation. However, the use of volitional control of affective states over an extended period of time can deplete cognitive resources (Baumeister et al., 1998; Englert, 2017). For athletes, this means that in highly emotional competitions and matches, cognitive resources can be exhausted after having been used permanently. As a result of depleted cognitive resources in the course of an emotionally draining match, there may be uncontrolled emotional outbursts and performance slumps due to decreased attention.

## Discussion

This paper established a new theoretical position on the role of affective processes in sport. In our view, the development of affective processes can be divided into six components that interact with each other and which constitute a cyclical process. From this understanding, some consequences arise that give this theoretical position an innovative value.

A key feature of the cycle of emotions is that the affective processes are highly dependent on the demands of the specific sport in question. As described above, the appraisals of the situation play a decisive role in setting affective processes in motion and deciding on which specific emotion comes into question. In contrast to other emotion theories in competitive sports, we do not postulate a general theory of emotion development in sports and a general relationship between emotions and performance. To understand the affective processes in a specific sport, one must understand the rules, the crucial situations as well as the social norms of the sport. For this reason, it is necessary to have precise knowledge of the respective sport in order to carry out both research as well as practical interventions successfully. It is therefore advisable to involve persons acquainted with the respective sport, such as coaches or ex-athletes.

Another consequence of the theory of the cycle of emotions is the importance of affect regulation skills (Fritsch and Jekauc, 2020). In our view, the use of affect regulation determines the extent to which the cycle of emotions develops and influences the performance of athletes. For sports psychology practice, this means that athletes should acquire certain skills for regulating their own affective states in the early stages of their development already. Currently, affect regulation skills develop more implicitly over the course of a career as athletes face and overcome various setbacks and challenges (Jones et al., 1994). In our opinion, this learning process can be made more systematic if the exercises for affect regulation are included in the athlete’s training plan and have a similar importance as the athletic training (Berking and Whitley, 2014).

This theory offers a new approach for classifying affect regulation and draws attention to several important affect regulation strategies in sports that are not considered in the currently dominant model of Gross (2014). For example, identifying and reflecting on one’s own goals, values, and expectations is an essential aspect of the triggering processes and, thus, affect regulation in applied sport psychology (Bisheff, 2009; Dohme et al., 2020). Here, we distinguish the triggering processes from the higher cognitive processes, which can make a crucial difference for athletes’ understanding of affect regulation. For example, regulating one’s own expectations and appraising the importance of the match is very important, especially in the run-up to the competition, because it determines the potential for triggering positive and negative affective states as well as for initiating the development of further affective processes. In contrast, strategies for regulating higher cognitive processes, such as imagery or self-talk, tend to be more important during the course of the match, when emotions have already elicited (Fritsch et al., 2020b).

Another key feature of the cycle of emotions as a theoretical position is the emphasis on the dynamics of affective processes in sports. It is probable that such affective processes are involved in the various highly dynamic phenomena in sports psychology, such as hot hand phenomenon, choking under pressure, psychological momentum, clutch performance, or collective team collapse. This idea is in line with studies that suggest that these psychological phenomena in sport might be caused by affective processes (Wang et al., 2004; Jones and Harwood, 2008; Wergin et al., 2018). However, more studies are needed to test these assumptions, namely whether affective processes cause these psychological phenomena. From a methodological point of view, these studies are very challenging, because they need to map the dynamics of affective processes. Observational studies focusing on several emotion components in real sport competitions are especially needed to understand the dynamics of affective processes and their influences on performance. Such analyses would require frequent or continuous measurement of indicators of affective states. Currently, however, research focuses on using one measure (Skoluda et al., 2012) or, at best, a few measures of affective processes (Vacher et al., 2019), but these represent only snapshots of this dynamic process (Sanchez et al., 2010). For this reason, the understanding of affective processes is rather limited.

This fact illustrates that the measurement of affective processes in sports is the main challenge. Currently, no measurement instrument exists that allows continuous recording of affective states during a competition. Therefore, we suggest that the measurement of affective processes should include multiple measurement tools that focus on multiple components of emotion at multiple time points. For example, during a competition, one could record physiological reactions (e.g., concentration of cortisol; Skoluda et al., 2012), expressive behaviors (e.g., through systematic observation; Moesch et al., 2015; Furley and Roth, 2021), and subjective experience (e.g., *via* a short questionnaire during a break; Jones et al., 2005). Such a multi-component assessment of affective states would help us understand the interaction of the components as well as the whole process of the development of affective states. In particular, it would be very interesting to know how the subjective emotional experience is related to the physiological or expressive component. Additionally, as a component of higher cognitive processes, one could capture self-talk after the match thanks to recorded scenes (cf. Fritsch et al., 2020b). Even though such a multimodal approach would be very costly, a deeper understanding of the affective processes during a match would increase. It would also allow for a better understanding of the interplay of the components of emotions.

## Conclusion

Within the context of this paper, a new theory about the role of emotions in sport is presented. The theory assumes six components of emotions interact with each other in a cyclical manner and constitute the dynamics of affective processes. It assumes that the peculiarities of each sport must be taken into account in order to understand the affective processes. Furthermore, this theoretical approach specifies the possible relationships between affective processes and performance, and provides a new taxonomy for affect regulation strategies. Consequences for research and practice are derived and discussed.

## Data Availability Statement

The original contributions presented in the study are included in the article/supplementary material, further inquiries can be directed to the corresponding author.

## Author Contributions

DJ and JF wrote the manuscript. AL edited the manuscript.

## Conflict of Interest

The authors declare that the research was conducted in the absence of any commercial or financial relationships that could be construed as a potential conflict of interest.

## Publisher’s Note

All claims expressed in this article are solely those of the authors and do not necessarily represent those of their affiliated organizations, or those of the publisher, the editors and the reviewers. Any product that may be evaluated in this article, or claim that may be made by its manufacturer, is not guaranteed or endorsed by the publisher.

## Footnotes

1 Component theories belong to the family of emotion theories that define emotions as a process involving changes in multiple subsystems or components (see Scherer, 2005). In general, component theories seek to break down a complex process into its elements and their interactions.

## References

[B1] Adie J. W. Duda J. L. Ntoumanis N. (2008). Achievement goals, competition appraisals, and the psychological and emotional welfare of sport participants. *J. Sport Exerc. Psychol.* 30 302–322. 10.1123/jsep.30.3.302 18648108

[B2] Arent S. M. Landers D. M. (2003). Arousal, anxiety, and performance: a reexamination of the inverted-U hypothesis. *Res. Q. Exerc. Sport* 74 436–444. 10.1080/02701367.2003.10609113 14768844

[B3] Augustine A. A. Hemenover S. H. (2009). On the relative effectiveness of affect regulation strategies: a meta-analysis. *Cogn. Emot.* 23 1181–1220. 10.1080/02699930802396556

[B4] Aviezer H. Trope Y. Todorov A. (2012a). Body cues, not facial expressions, discriminate between intense positive and negative emotions. *Science* 338 1225–1229. 10.1126/science.1224313 23197536

[B5] Aviezer H. Trope Y. Todorov A. (2012b). Holistic person processing: faces with bodies tell the whole story. *J. Pers. Soc. Psychol.* 103 20–37. 10.1037/a0027411 22352325

[B6] Banse R. Scherer K. R. (1996). Acoustic profiles in vocal emotion expression. *J. Pers. Soc. Psychol.* 70 614–636. 10.1037/0022-3514.70.3.614 8851745

[B7] Barrett L. F. Adolphs R. Marsella S. Martinez A. M. Pollak S. D. (2019). Emotional expressions reconsidered: challenges to inferring emotion from human facial movements. *Psychol. Sci. Public Interest* 20 1–68. 10.1177/1529100619832930 31313636 PMC6640856

[B8] Baumeister R. F. (1984). Choking under pressure: self-consciousness and paradoxical effects of incentives on skillful performance. *J. Pers. Soc. Psychol.* 46 610–620. 10.1037/0022-3514.46.3.610 6707866

[B9] Baumeister R. F. Bratslavsky E. Finkenauer C. Vohs K. D. (2001). Bad is stronger than good. *Rev. Gen. Psychol.* 5 323–370. 10.1037/1089-2680.5.4.323

[B10] Baumeister R. F. Bratslavsky E. Muraven M. Tice D. M. (1998). Ego depletion: is the active self a limited resource? *J. Pers. Soc. Psychol.* 74 1252–1265. 10.1037/0022-3514.74.5.1252 9599441

[B11] Baumeister R. F. Muraven M. Tice D. M. (2000). Ego depletion: a resource model of volition, self-regulation, and controlled processing. *Soc. Cogn.* 18 130–150. 10.1521/soco.2000.18.2.130

[B12] Baumeister R. F. Vohs K. D. Nathan Dewall C. Zhang L. (2007). How emotion shapes behavior: feedback, anticipation, and reflection, rather than direct causation. *Pers. Soc. Psychol. Rev.* 11 167–203. 10.1177/1088868307301033 18453461

[B13] Baumeister R. Heatherton T. Tice D. M. (1994). *Losing Control: How and Why People Fail at Self-Regulation.* San Diego, CA: Academic Press, Inc.

[B14] Beck A. T. (1976). *Cognitive Therapy and the Emotional Disorders.* Oxford: International Universities Press.

[B15] Berendt J. Uhrich S. (2018). Rivalry and fan aggression: why acknowledging conflict reduces tension between rival fans and downplaying makes things worse. *Eur. Sport Manag. Q.* 18 517–540. 10.1080/16184742.2018.1424226

[B16] Berking M. Whitley B. (2014). *Affect Regulation Training.* Heidelberg: Springer. 10.1007/978-1-4939-1022-9

[B17] Bisheff S. (2009). *Always Compete: An Inside Look at Pete Carroll and the USC Football Juggernaut.* New York, NY: St. Martin’s Press.

[B18] Blackledge J. T. Hayes S. C. (2001). Emotion regulation in acceptance and commitment therapy. *J. Clin. Psychol.* 57 243–255. 10.1002/1097-4679(200102)57:2<243::AID-JCLP9>3.0.CO;2-X 11180150

[B19] Buenemann S. Schweizer G. (2021). The reciprocal relationship between nonverbal behavior and sports performance in a cross-lagged panel model. *Psychol. Sport Exerc.* 55:101956. 10.1016/j.psychsport.2021.101956

[B20] Campos J. J. Frankel C. B. Camras L. (2004). On the nature of emotion regulation. *Child Dev.* 75 377–394. 10.1111/j.1467-8624.2004.00681.x 15056194

[B21] Carson H. J. Collins D. (2016). The fourth dimension: a motoric perspective on the anxiety–performance relationship. *Int. Rev. Sport Exerc. Psychol.* 9 1–21. 10.1080/1750984X.2015.1072231 26692896 PMC4662095

[B22] Carver C. S. Scheier M. F. (1990). Origins and functions of positive and negative affect: a control-process view. *Psychol. Rev.* 97 19–35. 10.1037/0033-295X.97.1.19

[B23] Carver C. S. Scheier M. F. (2001). *On the Self-Regulation of Behavior.* Cambridge: Cambridge University Press.

[B24] Cheng W.-N. K. Hardy L. Markland D. (2009). Toward a three-dimensional conceptualization of performance anxiety: rationale and initial measurement development. *Psychol. Sport Exerc.* 10 271–278. 10.1016/j.psychsport.2008.08.001

[B25] Clore G. L. Ortony A. (2008). “Appraisal theories: how cognition shapes affect into emotion,” in *Handbook of Emotions*, 3rd Edn, eds Lewis M. Haviland-Jones J. M. Barrett L. F. (New York, NY: The Guilford Press).

[B26] Coan J. A. (2010). Emergent ghosts of the emotion machine. *Emot. Rev.* 2 274–285. 10.1177/1754073910361978

[B27] Courts F. A. (1942). Relations between muscular tension and performance. *Psychol. Bull.* 39 347–368. 10.1037/h0060536

[B28] Cox R. H. Martens M. P. Russell W. D. (2003). Measuring anxiety in athletics: the revised competitive state anxiety inventory–2. *J. Sport Exerc. Psychol.* 25 519–533. 10.1123/jsep.25.4.519

[B29] Craft L. L. Magyar T. M. Becker B. J. Feltz D. L. (2003). The relationship between the competitive state anxiety inventory-2 and sport performance: a meta-analysis. *J. Sport Exerc. Psychol.* 25 44–65. 10.1123/jsep.25.1.44

[B30] Cunningham W. A. Zelazo P. D. (2007). Attitudes and evaluations: a social cognitive neuroscience perspective. *Trends Cogn. Sci.* 11 97–104. 10.1016/j.tics.2006.12.005 17276131

[B31] Cunningham W. A. Dunfield K. Stillman P. E. (2015). “Affect dynamics: iterative reprocessing in the production of emotional responses,” in *The Psychological Construction of Emotion*, eds Feldman Barrett L. Russell J. A. (New York, NY: The Guilford ress).

[B32] Cunnington R. Windischberger C. Deecke L. Moser E. (2003). The preparation and readiness for voluntary movement: a high-field event-related fMRI study of the Bereitschafts-BOLD response. *Neuroimage* 20 404–412. 10.1016/S1053-8119(03)00291-X 14527600

[B33] Dallas G. Charis S. Apostolos T. Dallas C. (2019). Competitive state anxiety and performance in young male artistic gymnasts. *Scie. Gymnast. J.* 11 299–306.

[B34] Darwin C. (1872). *The Expression of the Emotions in Man and Animals.* London: John Murray. 10.1037/10001-000

[B35] Davidson R. J. (2003). Affective neuroscience and psychophysiology: toward a synthesis. *Psychophysiology* 40 655–665. 10.1111/1469-8986.00067 14696720

[B36] Di Corrado D. Vitali F. Robazza C. Bortoli L. (2015). SELF-efficacy, emotional states, and performance in carom billiards. *Percept. Mot. Skills* 121 14–25. 10.2466/30.PMS.121c11x6 26226286

[B37] di Fronso S. Aquino A. Bondár R. Z. Montesano C. Robazza C. Bertollo M. (2020). The influence of core affect on cyclo-ergometer endurance performance: effects on performance outcomes and perceived exertion. *J. Sport Health Sci.* 9 578–586. 10.1016/j.jshs.2019.12.004 33308807 PMC7749253

[B38] Dohme L.-C. Bloom G. A. Piggott D. Backhouse S. (2020). Development, implementation, and evaluation of anathlete-informed mental skills training program for eliteyouth tennis players. *J. Appl. Sport Psychol.* 32 429–449. 10.1080/10413200.2019.1573204

[B39] Ekkekakis P. Petruzzello S. J. (2000). Analysis of the affect measurement conundrum in exercise psychology: I. Fundamental issues. *Psychol. Sport Exerc.* 1 71–88. 10.1016/S1469-0292(00)00010-8

[B40] Englert C. (2017). Ego depletion in sports: highlighting the importance of self-control strength for high-level sport performance. *Curr. Opin. Psychol.* 16 1–5. 10.1016/j.copsyc.2017.02.028 28813329

[B41] Eysenck M. W. Calvo M. G. (1992). Anxiety and performance: the processing efficiency theory. *Cogn. Emot.* 6 409–434. 10.1080/02699939208409696

[B42] Fletcher D. Hanton S. (2001). The relationship between psychological skills usage and competitive anxiety responses. *Psychol. Sport Exerc.* 2 89–101. 10.1016/S1469-0292(00)00014-5

[B43] Frijda N. H. (1986). *The Emotions.* New York, NY: Cambridge University Press.

[B44] Frijda N. H. (1988). The laws of emotion. *Am. Psychol.* 43 349–358. 10.1037/0003-066X.43.5.349 3389582

[B45] Fritsch J. Jekauc D. (2020). “Self-talk and emotion regulation,” in *Self-Talk in Sport*, eds Latinjak A. T. Hatzigeorgiadis A. (New York, NY: Routledge), 64–76. 10.4324/9780429460623-5

[B46] Fritsch J. Finne E. Jekauc D. Zerdila D. Elbe A.-M. Hatzigeorgiadis A. (2020a). Antecedents and consequences of outward emotional reactions in table tennis. *Front. Psychol.* 11:578159. 10.3389/fpsyg.2020.578159 33041951 PMC7522351

[B47] Fritsch J. Jekauc D. Elsborg P. Latinjak A. T. Reichert M. Hatzigeorgiadis A. (2020b). Self-talk and emotions in tennis players during competitive matches. *J. Appl. Sport Psychol.* 1–21. 10.1080/10413200.2020.1821406

[B48] Fritsch J. Redlich D. Latinjak A. Hatzigeorgiadis A. (2021). The behavioural component of emotions: exploring outward emotional reactions in table tennis. *Int. J. Sport Exerc. Psychol.* 1–19. 10.1080/1612197X.2021.1877324

[B49] Furley P. Roth A. (2021). Coding body language in sports: the nonverbal behavior coding system for soccer penalties. *J. Sport Exerc. Psychol.* 43 140–154. 10.1123/jsep.2020-0066 33730693

[B50] Furley P. Schweizer G. (2014). The expression of victory and loss: estimating who’s leading or trailing from nonverbal cues in sports. *J. Nonverb. Behav.* 38 13–29. 10.1007/s10919-013-0168-7

[B51] Furley P. Schweizer G. (2016). In a flash: thin slice judgment accuracy of leading and trailing in sports. *J. Nonverb. Behav.* 40 83–100. 10.1007/s10919-015-0225-5

[B52] Furley P. Schweizer G. (2020). “Body language in sport,” in *Handbook of Sport Psychology*, eds Tenenbaum G. Eklund R. C. (Hoboken, NJ: John Wiley & Sons), 1201–1219. 10.1002/9781119568124.ch59

[B53] Furley P. Dicks M. Memmert D. (2012). Nonverbal behavior in soccer: the influence of dominant and submissive body language on the impression formation and expectancy of success of soccer players. *J. Sport Exerc. Psychol.* 34 61–82. 10.1123/jsep.34.1.61 22356883

[B54] Furley P. Moll T. Memmert D. (2015). “Put your Hands up in the Air”? The interpersonal effects of pride and shame expressions on opponents and teammates. *Front. Psychol.* 6:1361. 10.3389/fpsyg.2015.01361 26441737 PMC4562262

[B55] Gardner F. L. Moore Z. E. (2007). *The Psychology of Enhancing Human Performance: The Mindfulness-Acceptance-Commitment (MAC) Approach.* New York, NY: Springer. 10.1891/9780826103369

[B56] Gardner F. L. Moore Z. E. (2020). “Mindfulness in sport contexts,” in *Handbook of Sport Psychology*, 4th Edn, eds Tenenbaum G. Eklund R. C. (Hoboken, NJ: Wiley), 738–750. 10.1002/9781119568124.ch35

[B57] Greenberg L. S. (2011). *Emotion-Focused Therapy.* Worcester, MA: American Psychological Association.

[B58] Greenlees I. Bradley A. Holder T. Thelwell R. (2005a). The impact of opponents’ non-verbal behaviour on the first impressions and outcome expectations of table-tennis players. *Psychol. Sport Exerc.* 6 103–115. 10.1016/j.psychsport.2003.10.002

[B59] Greenlees I. Buscombe R. Thelwell R. Holder T. Rimmer M. (2005b). Impact of opponents’ clothing and body language on impression formation and outcome expectations. *J. Sport Exerc. Psychol.* 27 39–52. 10.1123/jsep.27.1.39

[B60] Greenlees I. Leyland A. Thelwell R. Filby W. (2008). Soccer penalty takers’ uniform colour and pre-penalty kick gaze affect the impressions formed of them by opposing goalkeepers. *J. Sports Sci.* 26 569–576. 10.1080/02640410701744446 18344127

[B61] Gross J. J. (2014). “Emotion regulation: conceptual and empirical foundations,” in *Handbook of Emotion Regulation*, ed. Gross J. J. (New York, NY: Guilford Press), 3–20.

[B62] Gross J. J. Thompson R. A. (2006). “Emotion regulation: conceptual foundations,” in *Handbook of Emotion Regulation*, ed. Gross J. J. (New York, NY: The Guilford Press).

[B63] Hanin Y. L. (2007). “Emotions in sport: current issues and perspectives,” in *Handbook of Sport Psychology*, 3rd Edn, eds Tenenbaum G. Eklund R. C. (Hoboken, NJ: John Wiley & Sons Inc), 31–58. 10.1002/9781118270011.ch2

[B64] Hanton S. Fletcher D. Coughlan G. (2005). Stress in elite sport performers: a comparative study of competitive and organizational stressors. *J. Sports Sci.* 23 1129–1141. 10.1080/02640410500131480 16194989

[B65] Hardy L. (1990). “A catastrophe model of performance in sport,” in *Stress and Performance in Sport*, eds Jones J. G. Hardy L. (Oxford: John Wiley & Sons), 81–106.

[B66] Hashim H. A. Hanafi H. Yusof A. (2011). The effects of progressive muscle relaxation and autogenic relaxation on young soccer players’ mood states. *Asian J. Sports Med.* 2 99–105. 10.5812/asjsm.34786 22375225 PMC3289204

[B67] Hazell J. Cotterill S. T. Hill D. M. (2014). An exploration of pre-performance routines, self-efficacy, anxiety and performance in semi-professional soccer. *Eur. J. Sport Sci.* 14 603–610. 10.1080/17461391.2014.888484 24559097

[B68] Hill D. M. Shaw G. (2013). A qualitative examination of choking under pressure in team sport. *Psychol. Sport Exerc.* 14 103–110. 10.1016/j.psychsport.2012.07.008

[B69] Hill D. M. Hanton S. Matthews N. Fleming S. (2010). A qualitative exploration of choking in elite golf. *J. Clin. Sport Psychol.* 4 221–240. 10.1123/jcsp.4.3.221

[B70] Hodge K. Smith W. (2014). Public expectation, pressure, and avoiding the choke: a case study from elite sport. *Sport Psychol.* 28 375–389. 10.1123/tsp.2014-0005

[B71] Izard C. E. (1993). Four systems for emotion activation: cognitive and noncognitive processes. *Psychol. Rev.* 100 68–90. 10.1037/0033-295X.100.1.68 8426882

[B72] Jekauc D. Kittler C. Schlagheck M. (2016). Effectiveness of a mindfulness-based intervention for athletes. *Psychology* 8 1–13. 10.4236/psych.2017.81001

[B73] Jones G. (1995). More than just a game: research developments and issues in competitive anxiety in sport. *Br. J. Psychol.* 86 449–478. 10.1111/j.2044-8295.1995.tb02565.x 8542197

[B74] Jones G. Hanton S. Swain A. (1994). Intensity and interpretation of anxiety symptoms in elite and non-elite sports performers. *Pers. Individ. Differ.* 17 657–663. 10.1016/0191-8869(94)90138-4

[B75] Jones M. I. Harwood C. (2008). Psychological momentum within competitive soccer: players’ perspectives. *J. Appl. Sport Psychol.* 20 57–72. 10.1080/10413200701784841

[B76] Jones M. V. (2012). “Emotion regulation and performance,” in *The Oxford Handbook of Sport and Performance Psychology*, ed. Murphy S. M. (New York, NY: Oxford University Press), 154–172.

[B77] Jones M. V. Lane A. M. Bray S. R. Uphill M. Catlin J. (2005). Development and validation of the sport emotion questionnaire. *J. Sport Exerc. Psychol.* 27 407–431. 10.1123/jsep.27.4.407

[B78] Kachanathu S. J. Verma S. K. Khanna G. L. (2013). Effect of music therapy on heart rate variability: a reliable marker to pre-competition stress in sports performance. *J. Med. Sci.* 13:418. 10.3923/jms.2013.418.424

[B79] Kaufman K. A. Glass C. R. Arnkoff D. B. (2009). Evaluation of Mindful sport performance enhancement (MSPE): a new approach to promote flow in athletes. *J. Clin. Sport Psychol.* 3 334–356. 10.1123/jcsp.3.4.334

[B80] Kittler C. Gische C. Arnold M. Jekauc D. (2018). Der Einfluss eines achtsamkeitsbasierten trainings programms auf die emotions regulation von Sportlerinnen und Sportlern. *Z. Sportpsychol.* 25 146–155. 10.1026/1612-5010/a000242

[B81] Koole S. L. (2009). The psychology of emotion regulation: an integrative review. *Cogn. Emot.* 23 4–41. 10.1080/02699930802619031

[B82] Kopp A. Jekauc D. (2018). The influence of emotional intelligence on performance in competitive sports: a meta-analytical investigation. *Sports* 6:175. 10.3390/sports6040175 30551649 PMC6316207

[B83] Körner R. Petersen L.-E. Schütz A. (2019). Do expansive or contractive body postures affect feelings of self-worth? High power poses impact state self-esteem. *Curr. Psychol.* 40 4112–4124. 10.1007/s12144-019-00371-1

[B84] Kreibig S. D. (2010). Autonomic nervous system activity in emotion: a review. *Biol. Psychol.* 84 394–421. 10.1016/j.biopsycho.2010.03.010 20371374

[B85] Laborde S. Lautenbach F. Allen M. S. Herbert C. Achtzehn S. (2014). The role of trait emotional intelligence in emotion regulation and performance under pressure. *Pers. Individ. Differ.* 57 43–47. 10.1016/j.paid.2013.09.013

[B86] Landers D. M. (2007). “The arousal-performance relationship revisited,” in *Essential Readings in Sport and Exercise Psychology*, eds Smith D. Bar-Eli M. (Champaign, IL: Human Kinetics), 211–218.

[B87] Lane A. M. Beedie C. J. Jones M. V. Uphill M. Devonport T. J. (2012). The BASES expert statement on emotion regulation in sport. *J. Sports Sci.* 30 1189–1195. 10.1080/02640414.2012.693621 22709410

[B88] Lane A. Terry P. Karageorghis C. (1995). Antecedents of multidimensional competitive state anxiety and self-confidence in duathletes. *Percept. Mot. Skills* 80 911–919. 10.2466/pms.1995.80.3.911 7567411

[B89] Lautenbach F. Laborde S. (2016). “The influence of hormonal stress on performance,” in *Performance Psychology*, eds Raab M. Lobinger B. H. Hoffmann S. Pizzera A. Laborde S. (London: Elsevier), 315–328. 10.1016/B978-0-12-803377-7.00019-3

[B90] Lautenbach F. Laborde S. Mesagno C. Lobinger B. H. Achtzehn S. Arimond F. (2015). Nonautomated pre-performance routine in tennis: an intervention study. *J. Appl. Sport Psychol.* 27 123–131. 10.1080/10413200.2014.957364

[B91] Lazarus R. S. (1991). *Emotion and Adaptation.* New York, NY: Oxford University Press.

[B92] Lazarus R. S. (2000). How emotions influence performance in competitive sports. *Sport Psychol.* 14 229–252. 10.1123/tsp.14.3.229

[B93] Lazarus R. S. (2001). “Relational meaning and discrete emotions,” in *Appraisal Processes in Emotion*, eds Scherer K. Schorr A. Johnston T. (New York, NY: Oxford University Press).

[B94] Leahy R. L. Tirch D. Napolitano L. A. (2011). *Emotion Regulation in Psychotherapy: A Practitioner’s Guide.* New York, NY: Guilford press.

[B95] LeDoux J. (1998). *The Emotional Brain: The Mysterious Underpinnings of Emotional Life.* New York, NY: Simon and Schuster.

[B96] Lee H. Wäsche H. Jekauc D. (2018). Analyzing the components of emotional competence of football coaches: a qualitative study from the coaches’ perspective. *Sports* 6:123. 10.3390/sports6040123 30360530 PMC6316169

[B97] Lewis F. R. Knight C. J. Mellalieu S. D. (2017). Emotional experiences in youth tennis. *Psychol. Sport Exerc.* 29 69–83. 10.1016/j.psychsport.2016.12.003

[B98] Ludwig D. S. Kabat-Zinn J. (2008). Mindfulness in medicine. *JAMA* 300 1350–1352. 10.1001/jama.300.11.1350 18799450

[B99] Madden G. McGown C. (1988). The effect of hemisphericity, imagery, and relaxation on volleyball performance. *J. Hum. Mov. Stud.* 14 197–204.

[B100] Malouff J. M. Mcgee J. A. Halford H. T. Rooke S. E. (2008). Effects of pre-competition positive imagery and self-instructions on accuracy of serving in tennis. *J. Sport Behav.* 31 264–275.

[B101] Martens R. Vealey R. S. Burton D. (1990). *Competitive Anxiety in Sport.* Champaign, IL: Human Kinetics.

[B102] Martinent G. Ferrand C. (2009). A naturalistic study of the directional interpretation process of discrete emotions during high-stakes table tennis matches. *J. Sport Exerc. Psychol.* 31 318–336. 10.1123/jsep.31.3.318 19798996

[B103] Martinent G. Ledos S. Ferrand C. Campo M. Nicolas M. (2015). Athletes’ regulation of emotions experienced during competition: a naturalistic video-assisted study. *Sport Exerc. Perform. Psychol.* 4 188–205. 10.1037/spy0000037

[B104] Martinez S. G. (2017). *Aggression and Boxing Performance: Testing the Channeling Hypothesis with Multiple Statistical Methodologies.* Ph.D. thesi*s.* Dayton, OH: Wright State University.

[B105] Mccann P. Lavallee D. Lavallee R. (2001). The effect of pre-shot routines on golf wedge shot performance. *Eur. J. Sport Sci.* 1 231–240. 10.1080/17461390100071503

[B106] McGraw A. P. Mellers B. A. Tetlock P. E. (2005). Expectations and emotions of Olympic athletes. *J. Exp. Soc. Psychol.* 41 438–446. 10.1016/j.jesp.2004.09.001

[B107] McGregor H. Abrahamson E. (2000). The psychological effects of pre-competitive stress on elite divers—a review. *South Afr. J. of Psychol.* 30 38–44. 10.1177/008124630003000306

[B108] Mendes W. B. (2016). “Emotion and the automatic nervous system,” in *Handbook of Emotions*, 4th Edn, eds Barrett L. F. Lewis M. Haviland-Jones J. M. (New York, NY: The Guilford Press).

[B109] Mesagno C. Mullane-Grant T. (2010). A comparison of different pre-performance routines as possible choking interventions. *J. Appl. Sport Psychol.* 22 343–360. 10.1080/10413200.2010.491780

[B110] Mesagno C. Geukes K. Larkin P. (2015). “Choking under pressure: a review of current debates, literature, and interventions,” in *Contemporary Advances in Sport Psychology: A Review*, eds Mellalieu S. D. Hanton S. (New York, NY: Routledge).

[B111] Mitchell R. L. Phillips L. H. (2007). The psychological, neurochemical and functional neuroanatomical mediators of the effects of positive and negative mood on executive functions. *Neuropsychologia* 45 617–629. 10.1016/j.neuropsychologia.2006.06.030 16962146

[B112] Moesch K. Kenttä G. Bäckström M. Mattsson C. M. (2015). Exploring nonverbal behaviors in elite handball: how and when do players celebrate? *J. Appl. Sport Psychol.* 27 94–109. 10.1080/10413200.2014.953231

[B113] Moesch K. Kenttä G. Bäckström M. Mattsson C. M. (2018). Nonverbal post-shot celebrations and their relationship with performance in elite handball. *Int. J. Sport Exerc. Psychol.* 16 235–249. 10.1080/1612197X.2016.1216148

[B114] Moors A. De Houwer J. (2001). Automatic appraisal of motivational valence: motivational affective priming and Simon effects. *Cogn. Emot.* 15 749–766. 10.1080/02699930143000293

[B115] Moran A. P. (2016). *The Psychology of Concentration in Sport Performers: A Cognitive Analysis.* New York, NY: Psychology Press. 10.4324/9781315784946

[B116] Nguyen V. T. Breakspear M. Cunnington R. (2014). Reciprocal interactions of the SMA and cingulate cortex sustain premovement activity for voluntary actions. *J. Neurosci.* 34 16397–16407. 10.1523/JNEUROSCI.2571-14.2014 25471577 PMC6608485

[B117] Ochsner K. N. Gross J. J. (2005). The cognitive control of emotion. *Trends Cogn. Sci.* 9 242–249. 10.1016/j.tics.2005.03.010 15866151

[B118] Ochsner K. N. Silvers J. A. Buhle J. T. (2012). Functional imaging studies of emotion regulation: a synthetic review and evolving model of the cognitive control of emotion. *Ann. N. Y. Acad. Sci.* 1251 E1–E24. 10.1111/j.1749-6632.2012.06751.x 23025352 PMC4133790

[B119] Ortony A. Clore G. L. Collins A. (1990). *The Cognitive Structure of Emotions.* New York, NY: Cambridge University Press.

[B120] Parfitt G. Pates J. (1999). The effects of cognitive and somatic anxiety and self-confidence on components of performance during competition. *J. Sports Sci.* 17 351–356. 10.1080/026404199365867 10413262

[B121] Pineschi G. Di Pietro A. (2013). Anxiety management through psychophysiological techniques: relaxation and psyching-up in sport. *J. Sport Psychol. Act.* 4 181–190. 10.1080/21520704.2013.820247

[B122] Ramezanzade H. Arabnarmi B. (2011). Relationship of self esteem with forward head posture and round shoulder. *Proc. Soc. Behav. Sci.* 15 3698–3702. 10.1016/j.sbspro.2011.04.358

[B123] Rathschlag M. Memmert D. (2015). Self-generated emotions and their influence on sprint performance: an investigation of happiness and anxiety. *J. Appl. Sport Psychol.* 27 186–199. 10.1080/10413200.2014.974783 23535977

[B124] Robazza C. Bortoli L. (2007). Perceived impact of anger and anxiety on sporting performance in rugby players. *Psychol. Sport Exerc.* 8 875–896. 10.1016/j.psychsport.2006.07.005

[B125] Roemer L. Erisman S. M. Orsillo S. M. (2008). “Mindfulness and acceptance-based treatments for anxiety disorders,” in *Oxford Handbook of Anxiety and Related Disorders*, eds Antony M. M. Stein M. B. (New York, NY: Oxford University Press), 476–487. 10.1093/oxfordhb/9780195307030.013.0036

[B126] Roese N. J. Sherman J. W. (2007). “Expectancy,” in *Social Psychology: Handbook of Basic Principles*, eds Kruglanski A. Higgins E. (New York, NY: The Guilford Press), 91–115.

[B127] Rumbold J. L. Fletcher D. Daniels K. (2012). A systematic review of stress management interventions with sport performers. *Sport Exerc. Perform. Psychol.* 1 173–193. 10.1037/a0026628

[B128] Russell J. A. (2003). Core affect and the psychological construction of emotion. *Psychol. Rev.* 110 145–172. 10.1037/0033-295X.110.1.145 12529060

[B129] Russell J. A. (2009). Emotion, core affect, and psychological construction. *Cogn. Emot.* 23 1259–1283. 10.1080/02699930902809375

[B130] Russell J. A. (2015). “My psychological constructionist perspective, with a focus on conscious affective experience,” in *The Psychological Construction of Emotion*, eds Feldman Barrett L. Russell J. A. (New York, NY: Guilford Publications), 183–208.

[B131] Sanchez X. Boschker M. Llewellyn D. (2010). Pre-performance psychological states and performance in an elite climbing competition. *Scand. J. Med. Sci. Sports* 20 356–363. 10.1111/j.1600-0838.2009.00904.x 19486480

[B132] Sarason I. G. (1984). Stress, anxiety, and cognitive interference: reactions to tests. *J. Pers. Soc. Psychol.* 46 929–938. 10.1037/0022-3514.46.4.929 6737201

[B133] Scherer K. R. (2001). “Appraisal considered as a process of multilevel sequential checking,” in *Appraisal Processes in Emotion: Theory, Methods, Research*, eds Scherer K. R. Schorr A. Johnstone T. (New York, NY: Oxford University Press), 92–120.

[B134] Scherer K. R. (2005). What are emotions? And how can they be measured? *Soc. Sci. Inform.* 44 695–729. 10.1177/0539018405058216

[B135] Scherer K. R. (2009). The dynamic architecture of emotion: evidence for the component process model. *Cogn. Emot.* 23 1307–1351. 10.1080/02699930902928969

[B136] Seiler K. Schweizer G. Seiler R. (2018). Do the effects of nonverbal behaviour on team outcome confidence in team sports depend on the availability of additional performance information? *Psychol. Sport Exerc.* 36 29–40. 10.1016/j.psychsport.2017.12.007

[B137] Sève C. Ria L. Poizat G. Saury J. Durand M. (2007). Performance-induced emotions experienced during high-stakes table tennis matches. *Psychol. Sport Exerc.* 8 25–46. 10.1016/j.psychsport.2006.01.004

[B138] Sklett V. H. Lorås H. W. Sigmundsson H. (2018). Self-efficacy, flow, affect, worry and performance in elite world cup ski jumping. *Front. Psychol.* 9:1215. 10.3389/fpsyg.2018.01215 30065687 PMC6057433

[B139] Skoluda N. Dettenborn L. Stalder T. Kirschbaum C. (2012). Elevated hair cortisol concentrations in endurance athletes. *Psychoneuroendocrinology* 37 611–617. 10.1016/j.psyneuen.2011.09.001 21944954

[B140] Tomkins S. (1962). *Affect Imagery Consciousness: The Positive Affects.* New York, NY: Springer publishing company.

[B141] Uphill M. A. Jones M. V. (2007). Antecedents of emotions in elite athletes: a cognitive motivational relational theory perspective. *Res. Q. Exerc. Sport* 78 79–89. 10.1080/02701367.2007.10599406 17479577

[B142] Uphill M. A. Groom R. Jones M. V. (2014). The influence of in-game emotions on basketball performance. *Eur. J. Sport Sci.* 14 76–83. 10.1080/17461391.2012.729088 24533498

[B143] Uphill M. A. Mccarthy P. J. Jones M. V. (2009). “Getting a grip on emotion regulation in sport,” in *Advances in Applied Sport Psychology: A Review*, eds Mellalieu S. D. Hanton S. (New York, NY: Routledge), 162–194.

[B144] Vacher P. Filaire E. Mourot L. Nicolas M. (2019). Stress and recovery in sports: effects on heart rate variability, cortisol, and subjective experience. *Int. J. Psychophysiol.* 143 25–35. 31255740 10.1016/j.ijpsycho.2019.06.011

[B145] Vallacher R. R. Nowak A. (1997). The emergence of dynamical social psychology. *Psychol. Inq.* 8 73–99. 10.1207/s15327965pli0802_1

[B146] Vealey R. S. (1986). Conceptualization of sport-confidence and competitive orientation: preliminary investigation and instrument development. *J. Sport Exerc. Psychol.* 8 221–246. 10.1123/jsp.8.3.221

[B147] Wagstaff C. R. (2014). Emotion regulation and sport performance. *J. Sport Exerc. Psychol.* 36 401–412. 10.1123/jsep.2013-0257 25226609

[B148] Wang J. Marchant D. Morris T. Gibbs P. (2004). Self-consciousness and trait anxiety as predictors of choking in sport. *J. Sci. Med. Sport* 7 174–185. 10.1016/S1440-2440(04)80007-0 15362313

[B149] Weinberg R. S. Seabourne T. G. Jackson A. (1981). Effects of visuo-motor behavior rehearsal, relaxation, and imagery on karate performance. *J. Sport Exerc. Psychol.* 3 228–238. 10.1123/jsp.3.3.228

[B150] Weiner B. (1985). An attributional theory of achievement motivation and emotion. *Psychol. Rev.* 92 548–573. 10.1037/0033-295X.92.4.548 3903815

[B151] Weiner B. (1986). “Attribution, emotion, and action,” in *Handbook of Motivation and Cognition*, eds Sorrention R. Higgins E. (New York, NY: Guilford), 281–312.

[B152] Wergin V. V. Zimanyi Z. Mesagno C. Beckmann J. (2018). When suddenly nothing works anymore within a team–Causes of collective sport team collapse. *Front. Psychol.* 9:2115. 10.3389/fpsyg.2018.02115 30459685 PMC6232390

[B153] Wine J. (1971). Test anxiety and direction of attention. *Psychol. Bull.* 76 92–104. 10.1037/h0031332 4937878

[B154] Woodman T. Hardy L. (2001). “Stress and anxiety,” in *Handbook of Sport Psychology*, eds Singer R. N. Hausenblas H. A. Janelle C. M. (New York, NY: John Willey & Sons).

[B155] Woodman T. Hardy L. (2003). The relative impact of cognitive anxiety and self-confidence upon sport performance: a meta-analysis. *J. Sports Sci.* 21 443–457. 10.1080/0264041031000101809 12846532

[B156] Woodman T. Davis P. A. Hardy L. Callow N. Glasscock I. Yuill-Proctor J. (2009). Emotions and sport performance: an exploration of happiness, hope, and anger. *J. Sport Exerc. Psychol.* 31 169–188. 10.1123/jsep.31.2.169 19454770

[B157] Yerkes R. Dodson J. (1908). The relation of strength of stimulus to rapidity of habit-formation. *J. Comp. Neurol. Psychol.* 18 459–482. 10.1002/cne.920180503

